# *De Novo* Mutations in Moderate or Severe Intellectual Disability

**DOI:** 10.1371/journal.pgen.1004772

**Published:** 2014-10-30

**Authors:** Fadi F. Hamdan, Myriam Srour, Jose-Mario Capo-Chichi, Hussein Daoud, Christina Nassif, Lysanne Patry, Christine Massicotte, Amirthagowri Ambalavanan, Dan Spiegelman, Ousmane Diallo, Edouard Henrion, Alexandre Dionne-Laporte, Anne Fougerat, Alexey V. Pshezhetsky, Sunita Venkateswaran, Guy A. Rouleau, Jacques L. Michaud

**Affiliations:** 1 CHU Sainte-Justine Research Center, Montreal, Canada; 2 Division of Pediatric Neurology, Montreal Children's Hospital, Montreal, Canada; 3 Montreal Neurological Institute, McGill University, Montreal, Canada; 4 Division of Neurology, Children's Hospital of Eastern Ontario, Ottawa, Canada; 5 Department of Pediatrics and Department of Neurosciences, University of Montreal, Montreal, Canada; HudsonAlpha Institute for Biotechnology, United States of America

## Abstract

Genetics is believed to have an important role in intellectual disability (ID). Recent studies have emphasized the involvement of *de novo* mutations (DNMs) in ID but the extent to which they contribute to its pathogenesis and the identity of the corresponding genes remain largely unknown. Here, we report a screen for DNMs in subjects with moderate or severe ID. We sequenced the exomes of 41 probands and their parents, and confirmed 81 DNMs affecting the coding sequence or consensus splice sites (1.98 DNMs/proband). We observed a significant excess of *de novo* single nucleotide substitutions and loss-of-function mutations in these cases compared to control subjects, suggesting that at least a subset of these variations are pathogenic. A total of 12 likely pathogenic DNMs were identified in genes previously associated with ID (*ARID1B, CHD2, FOXG1, GABRB3, GATAD2B, GRIN2B, MBD5, MED13L, SETBP1, TBR1, TCF4, WDR45*), resulting in a diagnostic yield of ∼29%. We also identified 12 possibly pathogenic DNMs in genes (*HNRNPU, WAC*, *RYR2, SET, EGR1, MYH10*, *EIF2C1*, *COL4A3BP, CHMP2A, PPP1CB, VPS4A, PPP2R2B*) that have not previously been causally linked to ID. Interestingly, no case was explained by inherited mutations. Protein network analysis indicated that the products of many of these known and candidate genes interact with each other or with products of other ID-associated genes further supporting their involvement in ID. We conclude that DNMs represent a major cause of moderate or severe ID.

## Author Summary

Intellectual disability (ID) is the most frequent severe handicap of childhood. Several observations indicate that genetic factors explain a large fraction of cases with ID. We and others have recently found that *de novo* mutations (DNMs; genetic changes not transmitted from the parents) represent a common cause of ID. To further assess the contribution of DNMs to the development of ID, we interrogated virtually all the genes of the genome in 41 affected children with moderate or severe ID and in their healthy parents. In 12 of the cases, we identified disease-causing DNMs in genes known to be associated with ID, resulting in a molecular diagnostic yield of 29%. We also found 12 possibly disease-causing DNMs in genes that were not previously causally linked to ID. Interestingly, many of the genes with deleterious DNMs uncovered by this study encode proteins that interact with each other and affect specific processes in brain cells. In contrast, we did not identify any inherited mutations that could explain our cases. We conclude that DNMs play a predominant role in moderate or severe ID.

## Introduction

Intellectual disability (ID) is defined by significant impairment of cognitive and adaptive functions with onset before 18 years of age. It has an estimated worldwide prevalence of 1–3%, with moderate or severe forms of ID (IQ<50) affecting up to 0.5 % of the population in Western countries [1]. We and others have reported that *de novo* point mutations (including single nucleotide substitutions (SNVs) and small insertions/deletions, referred herein collectively as DNMs) play a significant role in the genetics of ID [2]–[5]. Similarly, DNMs were found to be implicated in the etiology of other neurodevelopmental disorders overlapping with ID, such as autism spectrum disorders (ASD), epileptic encephalopathy and schizophrenia [6]–[10]. DNMs represent the most extreme form of rare genetic variations; they are more deleterious, on average, than inherited variations because they have been subjected to less stringent evolutionary selection. Importantly, they provide a mechanism by which early-onset reproductively lethal diseases remain frequent in the population. This makes these mutations prime candidates for causing diseases that occur sporadically, and that decrease the reproductive fitness and incur a large degree of selection against phenotypes such as ID. Based on these considerations, we hypothesized that the contribution of DNMs is greater in more severe forms of ID. In order to explore this hypothesis, we performed high-depth exome sequencing in 41 trios consisting of individuals with moderate or severe ID and their healthy parents and assessed the contribution of DNMs to this condition.

## Results/Discussion

**Figure 1:**
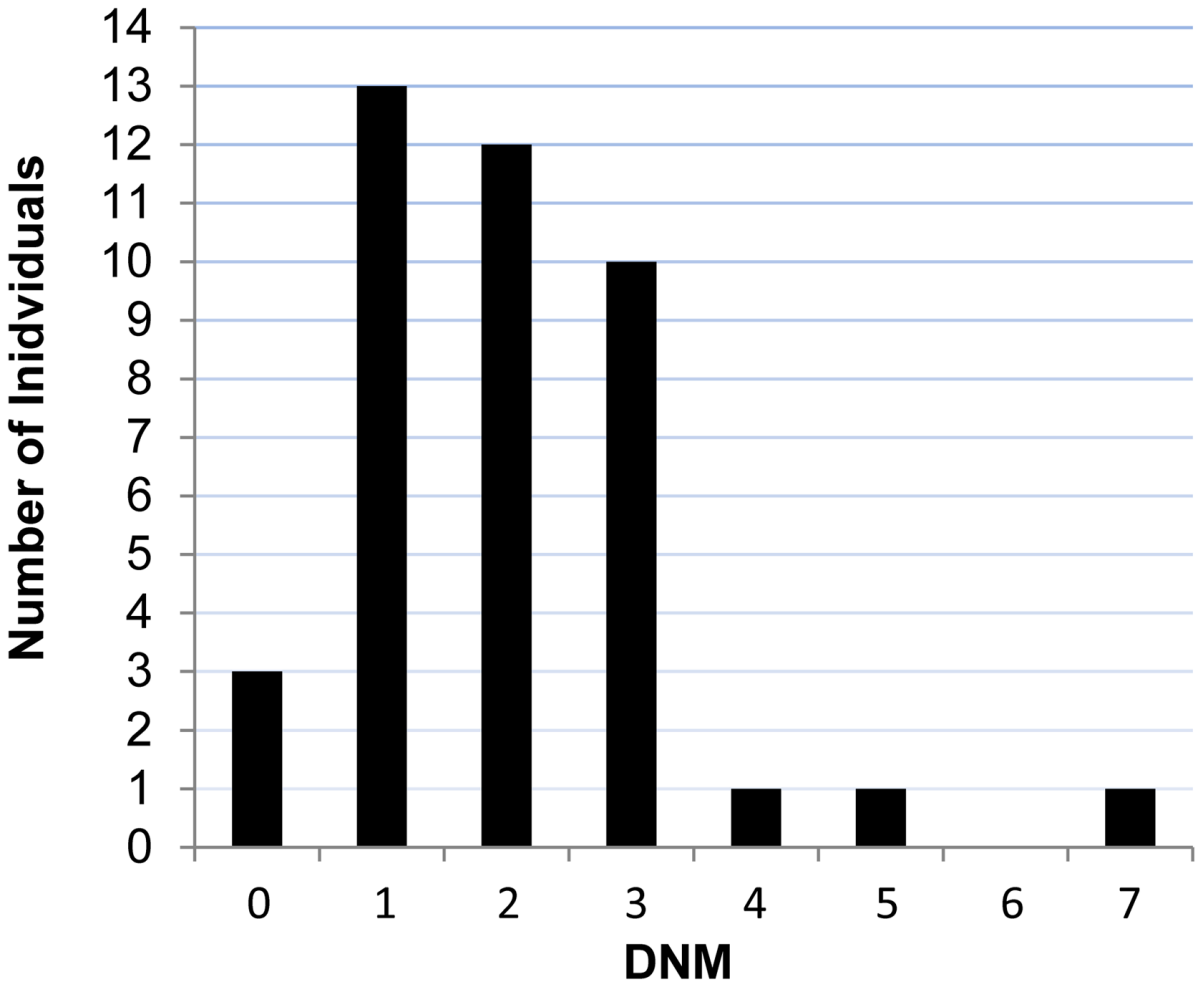
Number of DNMs per affected individual in each trio.

We performed exome sequencing in 41 individuals with ID and their unaffected parents. We identified a total of 83 putative DNMs in as many genes within both coding and consensus splice site sequences. Sanger sequencing confirmed 81 of these as *de novo* and 2 as inherited from one of the parents (Table S1). All of these DNMs were represented by ≥25% of reads, suggesting that they are unlikely to be associated with somatic mosaicism. The fact that the mutant and wild-type peaks on Sanger chromatograms were comparable in size is consistent with this conclusion. The average DNM rate per trio was 1.98, with only 3 trios containing no detectable DNMs (Figure 1). The observed *de novo* SNV rate in the consensus coding sequences (CCDS) was 1.56 events per trio or 2.58×10^−8^ per base per generation (64 SNVs in 2,477,702,175 CCDS bases sequenced at ≥10× in the 41 affected individuals), which is significantly higher than the expected population rate of 1.65×10^−8^ (*R* binomial test, *p* = 0.0007), or than the ones experimentally determined from exome sequencing studies in control trios (1.28×10^−8^ and 1.51×10^−8^) [2], [4]. Considering only *de novo* SNVs affecting the coding and the canonical splice sites (AG, GT at intronic positions −1/−2 and +1/+2 of the acceptor and donor splice sites, respectively), 73% were missense and 11% were nonsense and canonical splice site mutations. We found a significant excess of these *de novo* nonsense and splice site mutations in the probands of our cohort when compared to data from exome sequencing of 54 control trios with no family history of ID [4], [11] or of 593 quartets, including unaffected siblings of individuals with ASD (*R* binomial test, *p* = 0.0015 and *p* = 0.02, respectively) (Table 1) [7], [9], [10]. Such an excess of deleterious DNMs suggest that at least a subset of them are pathogenic.

**Table 1:** Distribution of the DNMs identified in this study and in controls.

DNM	This study (n = 41)	Trio controls (n = 54) refs [4], [11]	ASD unaffected siblings controls (n = 593) refs [7], [9], [10]
Missense	48	26	305
Synonymous	11	12	117
Nonsense	5	0	16
Canonical splice site	2	1	3
Consensus splice site*	1	0	NA
Frameshift	12	1	0
Inframe insertion/deletion	2	0	0
Total DNMs	81	40	441
Average DNM/trio	1.98	0.74	0.74
Average coding SNVs/trio	1.56	0.86	0.74
Total SNVs**	66	39	441
LoF SNVs	7	1	19
*p*-value (LoF SNVs) vs controls	-	**0.0015**	**0.02**

*canonical splice site variants not included.

**Consensus splice site variant not included.

NA, not applicable. LoF SNVs, nonsense and canonical splice site. Nominally significant *P* values (<0.05) calculated using an *R* exact binomial test.

Twelve DNMs were found in as many probands in genes previously associated with ID based on the documentation of deleterious DNMs in at least 4 unrelated individuals with similar phenotypes. Nine of these DNMs are Loss-of-Function (LoF) variants (nonsense, frameshift and canonical splice variants) and affect the following genes: *ARID1B* [OMIM 614556] [12], *CHD2* [OMIM 602119] [4], [13], *FOXG1* [OMIM 164874] [14], *GATAD2B* [OMIM 614998] [2], [15], *MBD5* [OMIM 611472] [9], [13], [16]–[18], *MED13L* [OMIM 608771] [7], [19]–[21], *SETBP1* [OMIM 611060] [4], [22], *TCF4* [OMIM 602272] [4], [23]–[25], and *WDR45* [OMIM 300526] [26], [27] (Tables 2 and 3). None of these 9 DNMs were found in public SNP databases. The phenotype of each of the probands is consistent with that of subjects previously described with mutations in these respective genes, with two exceptions (Text S1). Although truncating mutations in *CHD2* have been reported in individuals with epileptic encephalopathy [4], [6], [13], the individual described herein with a *CHD2* frameshift mutation has no history of epilepsy, suggesting that LoF mutations in *CHD2* are associated with greater clinical heterogeneity than initially expected. Another example of a gene associated with clinical heterogeneity in our dataset is *SETBP1*. Missense mutations clustering in a conserved 11-bp coding region of *SETBP1* have been reported to cause Schinzel-Giedon syndrome (OMIM 269150), a condition characterized by severe ID and specific craniofacial features [22]. In contrast, our case carried a *de novo* truncating mutation in *SETBP1* and showed moderate non-syndromic ID without the typical craniofacial features of Schinzel-Giedon syndrome. Recent studies reported a similar phenotype in individuals with a truncating mutation in *SETBP1* or microdeletions encompassing *SETBP1* [4], [28]. Collectively, these observations suggest that *SETBP1* haploinsufficiency results in a different phenotype than that induced by the missense mutations reported in Schinzel-Giedon syndrome, which presumably lead to a gain-of-function or a dominant negative effect [22]. We conclude that all of these 9 DNMs are likely to be pathogenic.

**Table 2:** Top risk DNMs identified in this study.

Individual	sex	Gene	Genomic change (hg19)	NCBI RefSeq.	AA	MutationType	Change; prediction (score)
289.143	F	*ARID1B*	chr6:157511198delC	NM_020732.3	2236	frameshift del	c.3716delC (Pro1239Hisfs*5)
1396.504	F	*CHD2*	chr2:93470514C>G	NM_001271.3	1828	nonsense	c.335C>G (p.Ser112*)
893.339	F	*FOXG1*	chr14:29236991delG	NM_005249.4	489	frameshift del	c.506delG (p.Gly169Alafs*23)
1907.666	F	*GATAD2B*	chr1:153785930T>C	NM_020699.2	593	CSS	c.1217-2A>G
79.65	M	*MBD5*	chr2:149221431_149221438delAAAAGCAT	NM_018328.4	1494	frameshift del	c.340_347del (p.Lys114Glyfs*35)
820.316	F	*MED13L*	chr12:116446509_116446510delCT	NM_015335.4	2210	frameshift del	c.1708_1709delCT (p.Ser570Phefs*27)
1861.653	M	*SETBP1*	chr18:42531126delC	NM_015559.2	1596	frameshift del	c.1821delC (p.Ser608Alafs*22)
1045.400	M	*TCF4*	chr18:52921925G>A	NM_001083962.1	667	nonsense	c.1153C>T (p.Arg385*)
1883.659	F	*WDR45*	chrX:48935736G>A	NM_007075.3	361	nonsense	c.C19T (p.Arg7*)
1843.647	M	*GABRB3*	chr15:26866506_26866507InsACC	NM_021912.4	473	insertion	c.413_415dupACC(p.Asn138_Arg139insHis); PVN (−12.3)
121.83	M	*TBR1*	chr2:162274305T>C	NM_006593.2	682	missense	c.811T>C (p.Trp271Arg); SIFT (0.00); PFF2 (1.0), PVN (−11.5)
838.321	M	*GRIN2B*	chr12:13720098C>T	NM_000834.3	1484	missense	c.2459G>A (p.Gly820Glu); SIFT (0.00). PFF2 (1.0), PVN (−7.5)
1464.524	M	*HNRNPU*	chr1:245027099G>A	NM_031844.2	825	nonsense	c.511C>T (p.Gln171*)
762.297	F	*WAC*	chr10:28824675_28824678delAGAG	NM_016628.4	647	frameshift del	c.263_266delAGAG (p.Glu88Glyfs*103)
341.162	M	*RYR2*	chr1:237995907G>A	NM_001035.2	4967	missense	c.14864G>A (p.Gly4955Glu); SIFT (0.00), PFF2 (1.00); PVN(−6.0)
1871.656	F	*MYH10*	chr17:8455445G>A	NM_001256012.1	2007	missense	c.838C>T (p.Arg280Cys); SIFT (0.00), PFF2 (1.00), PVN (−7.5)
702.278	F	*EIF2C1*	chr1:36359357G>A	NM_012199.2	857	missense	c.595G>A (p.Gly199Ser); SIFT (0.00), PFF2 (1.00), PVN (−5.2)
1312.477	M	*COL4A3BP*	chr5:74712811C>T	NM_001130105.1	752	missense	c.1111G>A (p.Gly371Arg); SIFT (0.002), PFF2 (0.97), PVN (−7.3)
115.81	M	*SET*	chr9:131456084_131456086delCTT	NM_001122821.1	290	frameshift del	c.699_701delCTT (p.Tyr233*)
670.267	F	*EGR1*	chr5:137803485_137803485insA	NM_001964.2	543	frameshift ins	c.1347_1348insA (p.Tyr450Ilefs*92)
1439.518	F	*PPP1CB*	chr2:29022094dupA	NM_206876.1	327	frameshift	c.909dupA (p.Tyr304Ilefs*19)
580.240	M	*CHMP2A*	chr19:59063688_59063688insG	NM_198426.2	222	frameshift	c.286_287insC (p.Asn96Thrfs*35)
1841.646	M	*PPP2R2B*	chr5:146070692C>G	NM_181678.2	501	missense	c.413G>C (p.Arg138Pro); SIFT (0.01); PFF2 (0.48); PVN (−4.9)
985.382	M	*VPS4A*	chr16:69353403_69353405delTCC	NM_013245.2	437	deletion	c.577_579delTCC (p.Ser193del); PVN (−12.3)

AA, total amino acids. All predictions by SIFT (http://sift.jcvi.org/), PFF2 (PolyPhen-2; http://genetics.bwh.harvard.edu/pph2/) and PROVEAN (PVN; (http://provean.jcvi.org/genome_submit.php) were damaging (scores indicated in parenthesis). CSS, Canonical splice site.

**Table 3:** Genes affected by predicted-damaging DNMs identified herein and their implication in ID.

Mutation type	ID-associated Genes with likely pathogenic DNMs	Candidate Genes with possibly pathogenic DNMs	Genes of unknown significance to IDψ
Missense	*GRIN2B, TBR1*	*MYH10, RYR2, EIF2C1, COL4A3BP, PPP2R2B*	*BCORL1, VIPR1, MTUS1, WDR33, R3HDM1, FBXO28, MAPKBP1, KCNH1*
Nonsense	*TCF4, CHD2, WDR45*	*HNRNPU*	
Canonical splice site*	*GATAD2B*		
Consensus splice site*			*GIT1*
Frameshift deletion	*MBD5, ARID1B, MED13L, FOXG1, SETBP1*	*WAC, SET*	
Inframe insertion	*GABRB3*		
Frameshift insertion		*EGR1, CHMP2A, PPP1CB*	
Inframe deletion		*VPS4A*	
Synonymous – splicing*			*NANS* **

All missense mutations were predicted damaging by SIFT and Polyphen-2. All in-frame deletions and insertions here were predicted damaging by PROVEAN (http://provean.jcvi.org/genome_submit.php).

*Predicted to affect splicing by both Human Splicing Finder (http://www.umd.be/HSF/) and Mutation Taster (http://www.mutationtaster.org/).

ψ Predicted-damaging DNMs present in cases with no likely or possibly pathogenic DNMs.

**Splicing defect verified by RT-PCR (Figure S1).

The three other DNMs in genes previously associated with ID include an in-frame insertion in *GABRB3* [OMIM 137192], a missense in *TBR1* [OMIM 604616] and a missense in *GRIN2B* [OMIM 138252] (Tables 2 and 3). All of these DNMs affect conserved residues and are predicted to be damaging. Moreover, none of them were found in public SNP databases. Damaging missense mutations in *GABRB3* have been previously documented in cases with ID and intractable epilepsy with various types of seizures [6]. Individual 1843.647 also showed ID and intractable epilepsy with a similar pattern of seizures as these cases (Text S1). DNMs in *TBR1* have been found in patients with ID and the variable presence of ASD or growth retardation [8], [9], [21], [29], [30]. Individual 121.83 displayed a phenotype similar to previously described cases, including ID, ASD and growth retardation (Text S1). Finally, DNMs in *GRIN2B* have been associated with ID of variable severity with or without ASD and epilepsy [2], [6], [31], [32]. Individual 838.321 showed severe ID, not walking and saying only one word at 16 years of age (Text S1). He has never had any seizures though his EEG revealed multifocal epileptic activity. Similar patterns of cognitive impairment were also reported in other patients with DNMs in *GRIN2B* [2], [32]. Interestingly, the mutation identified in our case affects a residue located in the ligand-binding domain of the protein, like previously reported *de novo* missenses in *GRIN2B* [32]. We conclude that these three DNMs are also likely to be pathogenic.

Among the remaining cases, 22 have predicted-damaging DNMs, including 7 LoF mutations (5 frameshifts, 1 nonsense, 1 consensus splice site), 13 missenses, 1 deletion, and 1 synonymous mutation whose predicted effect on splicing was confirmed by RT-PCR (Figure S1). Interestingly, deleterious DNMs in 6 of these genes (*HNRNPU* [OMIM 602869], *WAC* [OMIM 615049], *RYR2* [OMIM 180902], *MYH10* [OMIM 160776], *EIF2C1* [OMIM 606228], *COL4A3BP* [OMIM 604677]) have previously been reported in at least one individual with ID. We discuss hereafter the DNMs that we identified in these genes (Tables 2 and 3).

*HNRNPU* [OMIM 602869] codes for a highly conserved protein that binds RNAs and mediates different aspects of their metabolism and transport. Chromosome 1q44 microdeletions have defined a critical region associated with ID and seizures that encompasses *HNRNPU* as well as two other genes [33], [34]. Two truncating and one splice mutations in *HNRNPU* were subsequently identified in individuals with ID and seizures [6], [13], [24]. Two of these mutations occurred *de novo* whereas the origin of the other one was not elucidated. One of these individuals also showed ASD whereas the case with the splice mutation displayed syndromic features, including panhypopituitarism, bifid great toe and vertebral segmental defects. We identified an individual (1464.524) who carries a *de novo* truncating mutation (c.511C>T, p.Gln171*) in *HNRNPU*. This mutation is located in an upstream coding exon present in all isoforms, thus having the potential to induce nonsense mRNA mediated decay [35]. Moreover, inspection of the Exome Variant Server (EVS) database (6500 exomes) revealed no LoF variants in *HNRNPU*, indicating that haploinsufficiency of this gene is not tolerated. Our case displayed ID, epilepsy and ASD (Text S1), a phenotype that is similar to that of the other non-syndromic cases with DNMs in this gene, further supporting its involvement in ID.

*WAC* encodes a nuclear protein that interacts with RNF20/40 to regulate histone H2B ubiquinitation, chromatin organization, and gene transcription [36]. *De novo* microdeletions encompassing *WAC* and a nonsense DNM in *WAC* in individuals with severe ID were recently reported [2], [37]. Our subject (762.297) carries a truncating mutation in *WAC* (c.263_266del, p.Glu88Glyfs*103). This mutation is located in an upstream coding exon present in all isoforms. Inspection of the EVS database revealed no LoF variants in *WAC*. Individual 762.297 showed moderate ID without any distinguishing features on clinical examination and brain imaging, a phenotype that is consistent with that observed in the previously reported patient with a truncating mutation in this gene (Text S1) [2]. Our finding, thus, further supports the involvement of WAC in ID.

*RYR2* encodes the cardiac and brain-expressed calcium release channel ryanodine receptor 2. Mutations in *RYR2* are typically associated with exercise-induced ventricular and atrial arrhythmias. Virtually all reported mutations in *RYR2* are missenses or in-frame deletions that are believed to confer a gain of function, resulting in an increase of Ca+ release [38], [39]. We identified an individual (341.162) with ID, seizures, short stature and severe atrial arrhythmias (Text S1) who carries a predicted-damaging *de novo* missense mutation in *RYR2* (c.14864G>A, p.Gly4955Glu). Interestingly, 3 patients with seizures have previously been reported with DNMs in *RYR2*: 1) an individual with epileptic encephalopathy but presumably without a history of arrhythmia was recently found to carry a nonsense mutation (c.9568C>T, p.Arg3190*) in *RYR2* [6]; this DNM might not be disease-causing considering that the pathogenic impact of truncating mutations in *RYR2* remains unclear and that inspection of EVS revealed 5 different heterozygous LoF mutations in *RYR2*; 2) an individual with cognitive impairment, intractable seizures, short stature and subclinical ventricular tachycardia was found to carry a missense mutation (c.12563T>C, p.Leu4188Pro) [40]; and 3) an individual with intractable seizures but without cognitive impairment and arrhythmia was described with a missense mutation (c.14803G>A, p.Gly4935Arg) [41]. It is noteworthy that the mutation found in this latter individual is in close proximity to that of our subject, affecting a highly conserved C-terminal region of the protein. Interestingly, mice heterozygous for the missense mutation p.R2474S in *Ryr2* display generalized seizures and arrhythmias [42]. More recently, two brothers with ID, seizures and atrial arrhythmias were found to carry a missense mutation in *CLIC2* (OMIM 300138), which maps to the X chromosome [43]. CLIC2 is a negative regulator of RYR2. The mutation was shown to stimulate the release of Ca^2+^ by keeping the RYR2 channel in an open state, possibly due to a higher binding affinity for the RYR2 protein. The specificity of the phenotype observed in our subject and its similarity with that of other individuals with DNMs in *RYR2* or with the mutation in *CLIC2* suggest that the mutation identified herein may be causal.

*MYH10* encodes the non-muscle myosin heavy chain IIB that is critical for heart and brain development [44], [45]. Loss of *Myh10* function in mice results in embryonic lethality, hydrocephalus and neuronal migration defects but the cognitive and behavioural phenotype of heterozygous mice has not yet been reported. We identified a predicted-damaging *de novo* missense mutation (c.838C>T, p.Arg280Cys; individual 1871.656) in *MYH10*, affecting its conserved motor domain, whereas another group recently reported a *de novo* truncating mutation (c.2722G>T, p.Glu908*) in the same gene [46]. Both individuals displayed severe ID, microcephaly, and feeding difficulties as well as cerebral atrophy with increased intensities in bilateral basal ganglia and thalami on brain MRI (Text S1). The similarities between the phenotypes of these individuals raise the possibility that these mutations in *MYH10* are pathogenic. O'Roak et al. (2012) also reported a predicted-damaging *de novo* missense mutation (c.794A>G, p.Y265C; NM_001256012.1) in the motor domain of *MYH10*, in close proximity to the mutation identified herein, in a patient with ASD and moderate to severe ID. However no additional phenotypic data was available. Interestingly our patient with the *MYH10* mutation also displayed autistic features. Inspection of EVS for potential LoF mutations in *MYH10* showed the presence of a heterozygous frameshift deletion and a heterozygous splice site mutation. It is important to note, however, that these EVS variants were seen in single individuals and were not validated.

DNMs in *EIF2C1* and *COL4A3BP* have also been previously reported in single individuals with severe ID [2], [4]. For each of these genes, the phenotype of the affected individuals appears similar to that of our subjects (Text S1). However, because of the lack of specific clinical features in these individuals, the occurrence of DNMs in unrelated subjects does not readily indicate pathogenicity, especially in the case of missense mutations whose functional consequences are not validated.

**Figure 2:**
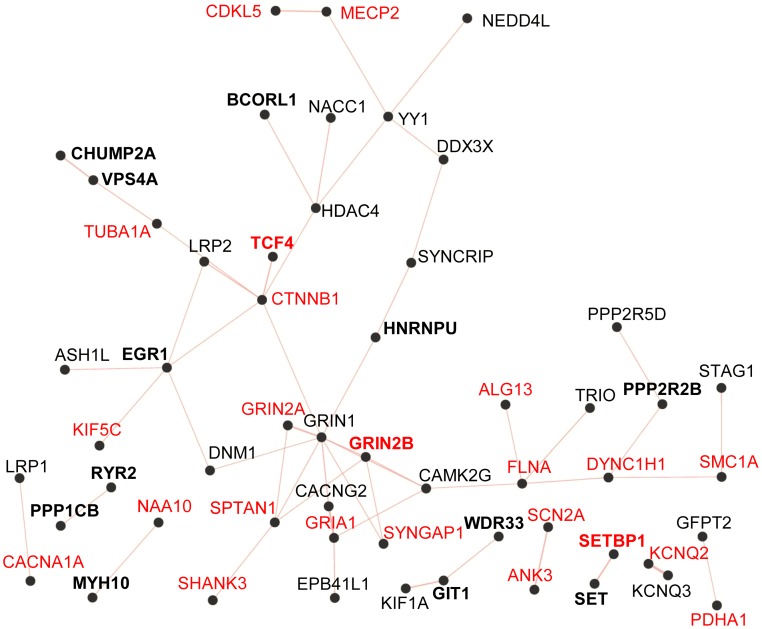
Physical protein-protein interaction network generated by GeneMANIA (http://www.GeneMANIA.org/; Gene Ontology molecular function based weighting). The Query genes included those listed in Table 3 from this study (in bold) and known and candidate ID genes reported with predicted-damaging DNMs from other studies (Table S2). Known ID genes are in red. The resulting network of 38 interconnected proteins was found to be enriched for proteins whose Gene Ontology molecular functions are implicated in the glutamate receptor signalling pathway (GRIN1, GRIN2A, GRIN2B, GRIA1, CACNG2, SHANK3; *FDR q*-value = 7.04e-6).

Among the remaining cases, we also identified 6 predicted-damaging DNMs in genes (*SET* [OMIM 600960], *EGR1* [OMIM 128990], *PPP1CB* [OMIM 600590], *CHMP2A* [OMIM 610893], *PPP2R2B* [OMIM 604325], and *VPS4A* [OMIM 609982]) that play biological functions relevant to ID (Table 2). Inspection of the EVS database revealed no LoF variants in these genes, with the exception of a single heterozygous variant in *PPP1C1B* (MAF = 1/12518) with a potential effect on splicing. In addition, some of these genes were found in proteomic studies to physically interact with the product of at least one ID-associated gene, further increasing the probability of their involvement in this disorder (see below and Figure 2). Each of these DNMs is discussed hereafter.

*SET* encodes a widely expressed multifunctional nuclear protein that affects pathways involved in ID, such as chromatin remodelling and gene transcription [47]. SET physically binds SETBP1 [48], whose disruption is known to cause severe ID (see above). In addition, recent studies indicate that SET directly interacts with MCPH1 (OMIM 607117) to ensure the proper temporal activation of chromosome condensation during mitosis [49]. Cells with *SET* knockdown exhibited abnormal condensed chromosomes similar to those observed in MCPH1-deficient fibroblasts. In addition, mutations that impair binding of MCPH1 to SET affect the ability of the former to rescue the abnormal chromosome condensation phenotype in fibroblasts from *Mcph1* mutant mice. Recessive mutations in *MCPH1* cause primary microcephaly, which is characterized by reduced brain size, without major structural abnormalities, and mild-to-moderate ID [50]. We identified a *de novo* deletion resulting in the creation of a premature stop codon in *SET* (c.699_701del, pTyr233*) in an individual (115.81) with congenital microcephaly, normal brain MRI, and moderate ID without any other distinguishing feature (Text S1). The functional relationship between MCPH1 and SET and the phenotypical similarities between cases with mutations in *MCPH1* and our subject suggest that the truncating DNM in *SET* may be pathogenic.

*EGR1* encodes a transcription factor that plays a key role in learning and memory [51]. We identified a *de novo* truncating mutation (c.1347_1348insA, p.Tyr450Ilefs*92) in *EGR1* in an individual (670.267) with severe non-syndromic ID and acquired microcephaly (Text S1). Mice harbouring a heterozygous deletion of *Egr1* showed synaptic plasticity, learning and memory impairments [52], [53]. Due to the prominent role of *EGR1* in learning and memory and the impact of its haploinsufficiency on cognition in mice, we postulate that the truncating DNM identified herein in *EGR1* may be pathogenic.

*PPP1CB*, which encodes a brain-enriched beta catalytic subunit of protein phosphatase 1 (PP1), and *PPP2R2B*, which encodes a neuron-specific B regulatory subunit of protein phosphatase 2 (PP2A), have been shown to regulate synaptic plasticity pathways [54], [55]. Individual 1439.518 carries a truncating mutation (c.909dupA, p.Tyr304Ilefs*19) in *PPP1CB*. This individual displayed severe ID, growth retardation and some dysmorphic features (Text S1). Individual 1841.646 carries a predicted-damaging missense mutation (c.413G>C, p.Arg138Pro) in *PPP2R2B*. This individual showed ID, intractable seizures and autistic features (Text S1). The pathogenic impact of these mutations remains uncertain at this point.

Among these candidate genes, *CHMP2A* and *VPS4A* are of special interest, as the proteins encoded by each are interacting partners. VPS4 ATPases play a critical role in the ESCRT pathway by recognizing membrane-associated ESCRT-III complexes and catalyzing their disassembly, a process that involves a direct interaction between CHMP2A and VPS4A [56]. The ESCRT-III pathway is involved in key cellular processes, including formation of endocytic multivesicular bodies, the abscission stage of cytokinesis, as well as centrosome and spindle maintenance [57]. Specific depletion of either CHMP2A or VPS4A proteins in cultured cells disrupts mitosis by inhibiting abscission and altering centrosome and spindle pole numbers [58]. We identified an individual (580.240) with a *de novo* frameshift insertion (c.286_287insC, p.Asn96Thrfs*35) in *CHMP2A* and another individual (985.382) with a predicted-damaging in-frame deletion (c.577_579delTCC, p.Ser193del) in *VPS4A* (Table 2). Both subjects showed severe ID as they were non-ambulatory and non-verbal at 4 years of age (Text S1). Our findings, thus, raise the possibility that components of the ESCRT-III complex maybe involved in ID.

To determine whether the genes identified here with predicted-damaging DNMs (likely/possibly pathogenic or of yet unknown significance to ID) (Table 3) encode proteins that are physically interconnected, we performed protein-protein interaction network analysis using GeneMANIA (http://www.GeneMANIA.org/) [59]. We also included in this analysis the known and candidate ID genes identified with predicted-damaging DNMs in other ID trio studies (Table S2) [2]–[6], [21]. This analysis showed that 11 out of the 24 proteins encoded by genes found herein with likely/possibly pathogenic DNMs interacted with either known or candidate ID genes, or with each other, further supporting their link to ID. Interestingly, we observed an enrichment for proteins implicated in glutamate receptor signaling pathways (*FDR q-value* = 7.04e-6) in the generated network (38 interconnected proteins) (Figure 2). Previous studies have shown an excess of functional DNMs over neutral ones in genes associated with glutamatergic systems in cases with non-syndromic ID, further supporting the critical involvement of this pathway in ID [3].

We also searched for the presence of rare inherited deleterious mutations (truncating, splicing, predicted-damaging missense and insertions or deletions) in genes associated with autosomal recessive or X-linked forms of ID, epilepsy or ASD (see Table S3 for the complete list of inherited rare variants in each proband). We identified only one case (692.274) that could potentially be explained by such mutations. This individual is hemizygous for a predicted-damaging missense (c.7949G>A [p.Arg2650His]; NM_031407.6) in the E3 ubiquitin ligase gene *HUWE1*, which is inherited from his healthy mother. Missense mutations in *HUWE1* have been associated with moderate to severe X-linked ID with normocephaly or macrocephaly [60]. Our case showed severe ID (non-verbal, non-ambulatory at 5 years of age) with congenital microcephaly. Because of these phenotypical differences, it is thus unclear whether this variation in *HUWE1* is pathogenic.

In summary, our trio exome sequencing study identified deleterious DNMs in genes previously causally linked to ID in 12 cases out of the 41 studied herein, resulting in a molecular diagnostic yield of 29%. Recently, de Ligt et al. (2012) and Rauch et al. (2012) performed trio exome sequencing in individuals with severe ID and obtained a diagnostic yield, based on the presence of predicted-damaging point mutations in currently known ID genes, of 20% and 35%, respectively [2], [4], [21]. Overall, the contribution of inherited autosomal or X-linked recessive mutations appears limited in the three cohorts. The study of Rauch et al (2012) and ours were intentionally centered on sporadic cases, which might have created a bias against inherited mutations. However, it is important to emphasize that most cases with moderate or severe ID are sporadic, at least in Western societies. de Ligt et al. (2012) observed a proportionally smaller number of DNMs in their cohort when compared to that of Rauch et al. (2012) and ours. This difference may be related to the use of a different sequencing technology, which is associated with a lower depth, possibly accounting for the lower diagnostic yield observed in this study. Indeed, exploration of a subset of unexplained cases from this cohort using whole-genome sequencing revealed additional pathogenic DNMs in known ID genes, bringing the point mutation molecular diagnostic yield in this cohort to 34% [21].

Our study also provides evidence for the potential pathogenicity of 12 additional DNMs in as many genes. Some of these genes represent strong candidates. For instance, both *HNRNPU* and *WAC* map to small critical regions associated with ID, which were defined by a series of microdeletions. *De novo* truncating mutations in each of these genes were previously described in cases with severe ID. We now report additional truncating DNMs in these genes in cases with similar phenotypes as those already published, further supporting their involvement in ID. Similarly, we and others have identified damaging DNMs in *RYR2* and *MYH10* in patients with similar features. Finally, we discovered a truncating DNM in *EGR1*, the haploinsufficiency of which affects learning and memory in mice. Although the characterization of additional cases will be needed to confirm the involvement of these candidate genes in ID, these results indicate that the contribution of DNMs to the pathogenesis of moderate or severe ID could be even greater than that suggested by the diagnostic rate observed in this study.

In conclusion, our study suggests that DNMs represent a predominant cause of moderate or severe ID. High-depth trio-based exome sequencing is an effective method to establish molecular diagnosis in such cases.

## Materials and Methods

### Study subjects and ethics statement

The cases reported here (18 males, 23 females) with moderate (n = 12) or severe (n = 29) ID were recruited at the Sainte-Justine Hospital (Montreal, Canada), after the approval of the ethics committee, and informed consent was obtained from each participant or legal guardian. Inclusion criteria for the probands were: 1) absence of a history of ID, epilepsy or ASD in first or second-degree relatives; 2) moderate or severe ID with or without epilepsy or autistic features; 3) absence of pathogenic copy number variants as revealed by array comparative genome hybridization performed on a clinical basis (using a 135k-feature whole-genome microarray (SignatureChip OS2.0 manufactured for Signature Genomic Laboratories (Spokane, WA, USA) by Roche NimbleGen, Madison, WI, USA); 4) absence of specific changes on brain imaging. The clinical description of the 41 affected individuals is summarized in Table S4. For cases with likely or possibly pathogenic variants, a more detailed clinical description can be found in Text S1.

### Exome capture and sequencing

Genomic DNA (3 µg) extracted from blood samples were used for exome capture and sequencing at the McGill University and Genome Quebec Innovation Center (Montreal, Quebec, Canada) using the Agilent SureSelect v4 exome capture kit, according to the manufacturer's recommendations, followed by 100 bp paired-end sequencing of each trio exomes on a single lane of the Illumina HiSeq2000.

### Data analysis

Exome sequence data processing, alignment (using a Burrows-Wheeler algorithm, BWA-mem), and variant calling were done according to the Broad Institute Genome Analysis Tool Kit (GATK v4) best practices (http://www.broadinstitute.org/gatk/guide/topic?name=best-practices), and variant annotation was done using Annovar [61]. The median coverage of the target bases was 135× with 95% of the target bases being covered ≥10×. We focused on variants affecting the exonic regions and consensus splice site sequences (defined herein as intronic bases up to positions −3 and +6 from the exon boundaries). Only variants whose positions were covered at ≥10× and supported by at least 4 variant reads constituting ≥20% of the total reads for each called position were retained. This typically yielded an average of ∼22,000 variants. This variant list was subsequently reduced to an average of ∼500 rare variants by filtering out those that are present in ≥0.5% of in-house exome data sets (n = 600) from unrelated projects, as well as variants present in the 1000 Genome or in the Exome Variant Server (EVS; http://evs.gs.washington.edu/EVS/) with minor allele frequencies (MAF) ≥0.5%. Putative DNMs (typically <10/exome) were then extracted from the rare variant list by further excluding those that were present in the exomes of the parents. The sequencing reads carrying putative DNMs were inspected visually in each trio, using the Integrative Genomics Viewer (IGV) [62], to exclude obvious false positives. All putative DNMs were validated by bidirectional Sanger sequencing in the corresponding trio.

## Supporting Information

Figure S1 Impact of the *NANS* synonymous *de novo* mutation in exon 4, c.603G>A (p. = ) (NM_018946.3), identified in patient 143.91 on exon splicing.
(DOC)

Table S1 Confirmed DNMs identified in this study.
(XLSX)

Table S2 Genes included in the physical protein-protein interaction network analysis.
(XLSX)

Table S3 Inherited rare variations identified in the 41 probands of this study.
(XLSX)

Table S4 Clinical phenotypes of the 41 affected individuals.
(XLSX)

Text S1 Detailed clinical description of the patients with likely and possibly pathogenic DNMs identified in this study.
(DOCX)
