# Supplementary material for: De Novo Mutations in Moderate or Severe Intellectual Disability
Source: PLoS Genet. 2014 Oct 30;10(10):e1004772. doi: 10.1371/journal.pgen.1004772 (PMC4214635; doi:10.1371/journal.pgen.1004772)
Supplement: Figure S1 — Impact of the NANS synonymous de novo mutation in exon 4, c.603G>A (p. = ) (NM_018946.3), identified in patient 143.91 on exon splicing. (DOC) [file pgen.1004772.s001.doc]

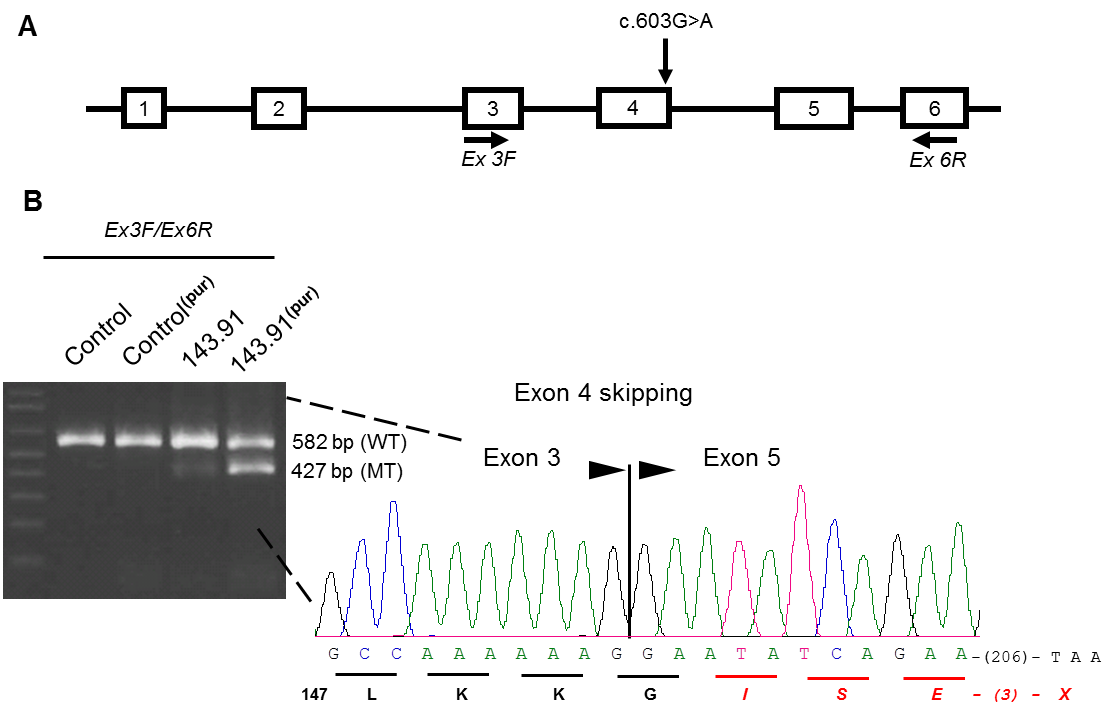


**Figure S1**. Impact of the *NANS* synonymous *de novo* mutation c.603G>A (p.=) (NM_018946.3) identified in patient 143.91 on exon splicing. **A)** Schematic representation of the *NANS* (NM_018946.3) exons/introns flanking the mutation showing the position of the c.603G>A mutation at the exon-4/intron-4 boundary and the PCR primers flanking it. **B)** Effect of c.603G>A on exon splicing. Total RNAs from patient 143.91 and an unrelated control subject were extracted (Trizol reagent; Invitrogen) from lymphoblastoid cell line cultures, either treated with 300 µg/ml of puromycin (pur) for 6 hours or not. One microgram of DNAse-treated total RNA from each sample was used to generate complementary DNA (cDNA) using the MMLV Reverse transcriptase (Invitrogen, Carlsbad, California). The resulting cDNAs were PCR-amplified with *NANS*-specific primers flanking exon 4, with a forward primer in exon 3 (Ex3F: 5’-CTGCATGAACTGAATGTTCCA-3’) and a reverse primer in exon 6 (Ex6R: 5’- CTTCACGGTGAGCATGTCC-3’). Resulting PCR products obtained from the patient’s sample were subcloned into TOPO-TA PCR2.1 (Invitrogen) and Sanger sequenced. Left panel shows agarose gel electrophoresis of the reverse transcription (RT)-PCR products obtained from the control and the patient samples. The cDNA fragment covering exons 3-6 of *NANS* was amplified by RT-PCR. The expected 582-bp wild-type (WT) PCR product was observed from the control and the patient samples, whereas a smaller mutant product 427-bp (MT) was only detected from the patient cDNAs. The intensity of this smaller product was higher after treatment of the patient’s cells with puromycin (143.91^(pur)^), which inhibits protein translation, indicating some amount of non-sense mediated decay. Sanger sequencing of the MT fragment from the patient indicated a *NANS* transcript lacking exon-4, resulting in a frameshift starting at amino acid 151 and that extends for 6 amino acids followed by a stop codon. The chromatogram showing the skipping of exon-4 in the patient (MT band) and the corresponding amino acids as well as the resulting frameshift (highlighted) are illustrated.
