# Supplementary material for: De Novo Mutations in Moderate or Severe Intellectual Disability
Source: PLoS Genet. 2014 Oct 30;10(10):e1004772. doi: 10.1371/journal.pgen.1004772 (PMC4214635; doi:10.1371/journal.pgen.1004772)
Supplement: Text S1 — Detailed clinical description of the patients with likely and possibly pathogenic DNMs identified in this study. (DOCX) [file pgen.1004772.s006.docx]

**Text S1.** Detailed clinical description of the patients with likely and possibly pathogenic DNMs identified in this study

**Case 289.143. Mutation c.3716delC (Pro1239Hisfs*5) in *ARID1B*.** The patient is a 10-year-old girl. She is the product of an uncomplicated pregnancy and delivery. She was noted to have bilateral hip subluxation at birth and a small atrial septal defect that spontaneously closed. She also has a scoliosis requiring the use of a corset. She was globally delayed. She started walking with support at the age of 20 months, and independently at 3 years. Her first words were at the age of two. She can speak in complete sentences. She can also do simple additions, multiplications and divisions. She had heterogeneous scores on the WISC-III, with overall moderate ID score. The patient had 3 partial complex seizures at the age of 8 years, for which she was started on carbamazepine and has not had any recurrence. On examination at 5 years and 9 months of age, height was 106.5 cm (10^th^ percentile), weight 19.9 kg (50^th^ percentile) and head circumference 51 cm (50^th^ percentile). She had dysmorphic features including of down-slanting palpebral fissures, low-set ears, coarse features with full lips, broad nasal root, bulbous nose and long philtrum. She has large distal phalanges except for the 5^th^ digit bilaterally. Clinically, these features are consisting with Coffin-Siris syndrome. Neurological exam revealed axial and peripheral hypotonia with normal strength. Reflexes were normal in the upper limbs but brisk in the lower limbs. Investigations included karyotyping, chromosomal microarray, metabolic studies (plasma lactate and ammonia measurements, plasma amino acids and urine organic acids chromatographies) and *DM1* triplet testing for myotonic dystrophy type 1. Brain MRI and MR spectroscopy were unremarkable.

**Case 1396.504. Mutation c.335C>G (p.Ser112*) in *CHD2*.** This girl is currently 5.5 years old. Perinatal history was unremarkable. She was referred to medical attention for global developmental delay and microcephaly. She walked at the age of 20 months. She started to say words at 2.5 years. At the age of 5, she can run, ride the tricycle, and jump. She makes short complete sentences and understands simple commands. She knows a few colours but she does not recognize any numbers or letters. She is sociable. She is impulsive and has attention deficit. She has not had any seizures. On examination, height is 108.3 cm (90-97^th^ percentile), weight 17.2 kg (25-50^th^ percentile) and head circumference 46 cm (<3^rd^ percentile). She is not dysmorphic. General and neurologic examinations are unremarkable. Karyotyping, chromosomal microarray, Fragile X testing, *SYNGAP1* mutation analysis, and metabolic studies (plasma ammonia and lactate measurements, urine creatine/guanidinoacetate and purines/pyrimidines measurements and plasma amino acids and urine organic acids chromatographies) were normal. Brain CT scan at the age of 13 months was normal.

**Case 893.339. Mutation c.506delG (p.Gly169Alafs*23) in *FOXG1*.** This is a 22-year-old female with severe ID. On prenatal ultrasounds, a deceleration of her head growth was noted and the patient was microcephalic at birth with a head circumference of 31 cm. Parents were concerned regarding developmental delay within the first few months of life. She presented with seizures at the age of 9 months. She still has intractable epilepsy, with primarily partial complex seizures. She currently takes valproic acid, clonazepam and clobazam. She continues to have on average 3 seizures a week. She is severely delayed. She is wheel-chair bound and is unable to sit independently. She can reach and transfer objects. She is nonverbal. She has a social smile. She can only eat purees. She has a scoliosis. On exam at the age of 22 years, her head circumference is 47cm (<2^nd^ percentile). She has malar hypoplasia. She also has bilateral strabismus. Her eye contact is not sustained. She also has a movement disorder with choreoathetotic and dystonic movements of the limbs, especially the upper limbs. Her tone is increased and her reflexes are brisk. Karyotyping, chromosomal microarray, *MECP2* mutation analysis, and metabolic studies (plasma ammonia and lactate measurements, CSF lactate measurement, plasma amino acids and urine organic acids chromatographies) were normal. Brain MRI performed at 13 months of age was normal.

**Case 1907.666. Mutation c.1217-2A>G in *GATAD2B.*** This 5-year-old girl was born following an uneventful pregnancy and delivery. She had global developmental delay. She started to walk at 22 months. At 5 years, she could not run and would fall easily. She could not jump. She has no words but speaks in jargon. She does not communicate with signs nor pictograms. She understands some simple commands. She can point to a few body parts. She can scribble but does not draw a circle. She has a tendency to have tamper tantrums when upset. She has autistic traits but she does not satisfy the criteria for a diagnosis of autism on formal evaluation. At 5 years, she was diagnosed with epilepsy, presenting with absence seizures and was treated with leviteracetam. EEG revealed generalized epileptic activity. On exam at the age of 5 years, her weight was 15 kg (3-15^th^ percentile), height 96.7 c (<3^rd^ percentile). Her head circumference is at the 50^th^ percentile. She is hyperteloric, has epicanthal folds, some prognathism and a broad mouth. She has mildly lax ligaments. Her general and neurologic exams were unremarkable. Chromosomal microarray, *MECP2* mutation analysis, methylation studies of the genomic region associated with Angelman syndrome, and Fragile X testing were normal. Brain MRI at the age of 2 was normal, except for myelination delay.

**Case 79.65. Mutation c.340_347del (p.Lys114Glyfs*35) in *MBD5*.** This patient is currently 14 years old. He was brought to medical attention at the age of 5 months for severe hypotonia and developmental delay. He developed epilepsy in the first year of life, which became intractable. He has multiple seizure types that include generalized tonic, tonic-clonic and atypical absence. He currently has 2 seizures per day. He takes leviteracetam, clobazam and topiramate. EEGs revealed multifocal epileptic activity with slow spike and wave with bifrontal predominance and disturbance of the background. He has severe intellectual disability. He is wheelchair bound, does not sit and can support his head for short periods of time. He does not reach for objects. He does not visually fix and follow. He has no words, and does not recognize his name. During infancy, his neurologic exam revealed severe hypotonia. On his latest examination, he is spastic in his four limbs. He is normocephalic, with a head circumference at the 5^th^ percentile. Karyotyping, chromosomal microarray and metabolic studies (plasma lactate, ammonia, and very long chain fatty acids measurements, plasma amino acids and urine organic acids chromatographies, isoelectric focusing of serum transferrin, urine purines/pyrimidines measurements, plasma acylcarnitines profile) were normal. Muscle biopsy was normal. Initial cerebral MRI revealed myelination delay, thin corpus callosum and mild ventriculomegaly. Follow-up MRI revealed progression of myelination.

**Case 820.316. Mutation c.1708_1709delCT (p.Ser570Phefs*27) in *MED13L.*** This female was the product of an uncomplicated pregnancy and delivery. She was investigated for global developmental delay. She walked at the age of 3 years. At the age of 5 years, she could make short, incomplete sentences. She could understand simple, familiar commands. She could recognize colours and count to 20. She did not recognize the letters. She could get dressed by herself. She was overweight, and her weight was at the 97^th^ percentile for age since 4 months. She has strabismus. Neuropsychology testing revealed moderate ID. She has not had any seizures. She has no behavioural problems. At the age of 5 years, her weight was 25.7 kg (97^th^ percentile), her height was 110.7 kg (75^th^ percentile) and her head circumference was 53 cm (75^th^ percentile). Chromosomal microarray, karyotype, Fragile X testing, and metabolic studies (plasma lactate, ammonia, and very long chain fatty acids measurements, plasma amino acids and urine organic acids chromatographies, isoelectric focusing of serum transferrin, urine creatine/guanidinoacetate and purines/pyrimidines measurements) were normal. Brain CT scan performed at the age of 2 years showed mildly increased extra-axial CSF spaces.

**Case 1861.653. Mutation c.1821delC (p.Ser608Alafs*22) in *SETBP1.*** This male patient is currently 6.5 years old. He has ID. He walked at 25 months. At the age of 6.5 years, he could run, jump and go up the stairs alternating with support. He makes 2 to 3-word sentences but his pronunciation is difficult. He understands simple sentences and has difficulty with abstract concepts. He is treated with lisdexamphetamine (Vyvanse) for attention deficit and hyperactivity. He is sociable. He has never had any seizures. At 6.5 years, his height was 107.4 cm (3^rd^ percentile), his weight 16.8 kg (3^rd^ percentile) and head circumference 49.5 cm (3^nd^ percentile). He has no dysmorphic features. His general and neurologic examinations are unremarkable. Chromosomal microarray, karyotype, metabolic studies (plasma lactate, ammonia, and very long chain fatty acids measurements, plasma amino acids and urine organic acids chromatographies, isoelectric focusing of serum transferrin), methylation studies of the genomic region associated with Angelman syndrome, *UBE3A* mutation analysis were normal. Brain MRI at the age of 2 was normal except for myelination delay.

**Case 1045.400. Mutation c.1153C>T (p.Arg385*) in *TCF4*.** This boy was born following an unremarkable pregnancy and delivery. He had severe global developmental delay. At the age of 6 years, he could sit alone, crawl, take steps with a walker but was unable to stand without support. He did not say any words and did not seem to understand. He did not have a pincer grasp. He was depended on all activities of daily living. He received salivary gland botox injections for hypersalivation. His breathing was normal and there was no apnea. He has had only one seizure, and he is not treated with any anticonvulsants. Electroencephalogram was normal. At the age of 6 years, his weight was 21.4 kg (50^th^ percentile), height 112.5 cm (10-25%) and head circumference 50.5cm (50^th^ percentile). He has minor dysmorphisms, including a wide mouth, bilateral single palmar creases, bilateral clinodactyly and overlapping 2^nd^ toes. He has central hypotonia and mild increased tone in the limbs. Clinically, these features are consisting with Pitt-Hopkins syndrome. Chromosomal microarray, methylation study of the genomic region associated with Angelman syndrome, Fragile X testing, *MECP2* mutation analysis, and metabolic studies (plasma lactate, ammonia, and very long chain fatty acids measurements, plasma amino acids and urine organic acids chromatographies, isoelectric focusing of serum transferrin, urine creatine/guanidinoacetate and purines/pyrimidines measurements) were normal. Brain MRI performed at the age of 1 revealed increased T2 and FLAIR signal in the periventricular regions, a thin corpus callosum, and myelination delay. Brain MR spectroscopy was normal.

**Case 1883.659. Mutation c.C19T (p.Arg7*) in *WDR45*.** This patient is a 4-year-old girl. Pregnancy and perinatal history were unremarkable. She is severely developmentally delayed. She started to sit at the age of 7,5 months. At the age of 4 years, she is unable to walk but has started to stand briefly without support. She can transfer objects but does not use a spoon or scribble with a pencil. She does not have any words and seem to understand only very limited routine words. Initially, she was very hypotonic but has developed spasticity in her limbs. She developed epilepsy at the age of 3.5 years with atonic seizures. She is currently taking leviteracetam with good seizure control. EEG showed generalized epileptiform activity with irregular spike and polyspike, and some photosensitivity. On exam at the age of 4 years, her head circumference was 48.2 cm (25^th^ percentile). There is a decrease in the rare of growth of her head (at the 85-98^th^ percentile at birth). She is not dysmorphic. She drools. She is very smiley. She has mild spasticity in the limbs, but no extrapyramidal signs nor any movement disorder. Chromosomal microarray, *MECP2* mutation analysis, methylation study of the genomic region associated with Angelman syndrome, and metabolic studies (plasma lactate and ammonia measurements, plasma amino acids and urine organic acids chromatographies, urine purines/pyrimidines measurements, and isoelectric focusing of serum transferring) were normal. Brain MRI was normal and there were no basal ganglia abnormalities.

**Case 1843.647. Mutation c.413_415dupACC (p.Asn138_Arg139insHis) in *GABRB3.*** This patient is a 2.5 year old boy. Pregnancy and delivery were unremarkable. He presented with seizures at the age of 2 months. He had several types including myoclonic seizures, head drops and partial complex seizures. EEG at the time revealed multifocal epileptic activity and severe disturbance of the background that evolved to a modified burst-suppression pattern. His seizures were initially difficult to control and they were resistant to vigabatrin, ACTH and Topiramate. He responded very well to leviteracetam, which was added at the age of 4.5 months. He is currently well-controlled on topiramate and leviteracetam. He also has global developmental delay. He started to walk at the age of 22 months. At the age of 2 years, he could climb stairs with support and was starting to use a spoon to feed. He could wave bye-bye and say approximately 10 words. He would repeat a lot. He had poor eye contact and some mild autistic features. On examination at the age at 2, his head circumference was 50,2cm (50^th^ percentile). He is not dysmorphic. His neurologic examination is normal. Microarray revealed a 0.158Mb duplication on chromosome Yq11.22 that was paternally inherited and deemed to be of no clinical significant. *STXBP1* mutation analysis and metabolic studies (isoelectric focusing of serum transferrin, plasma biotinidase activity levels, plasma amino acids and urine organic acids chromatographies, urine creatine/guanidinoacetate, purines/pyrimidines and alpha aminoadipic semialdehyde measurements, plasma very long chain fatty acids and CSF neurotransmitters measurements, plasma acylcarnitine profile) were normal. Brain MRI was unremarkable.

**Case 838.321. Mutation c.2459G>A (p.Gly820Glu) in *GRIN2B.*** This male patient was born following an unremarkable pregnancy and delivery. He had severe global developmental delay and failure to thrive. At the most recent evaluation at the age of 16 years, he was wheel-chair dependent and could not sit independently. He had no words except for “no”. He could only understand a few simple commonly-used commands. He can reach for objects but has no pincer grasp. He was dependent on all activities of daily living. He was dysphagic and ate only pureed foods. He has never had any seizures though his EEG revealed multifocal epileptic activity. He is microcephalic. His hearing and vision were normal. On exam, at the age of 16 years, his head circumference is 51.5 cm (<2^nd^ percentile). He has no dysmorphic features. He has a left eye exotropia. There is no nystagmus and extra-ocular movements are full. There is significant decreased muscle bulk. Tone and strength were normal. There is no evidence of clear dysmetria or ataxia. Karyotyping, chromosomal microarray, plasma lactate and ammonia measurements were normal. Brain MRI at the age 7 years revealed some left temporal lobe atrophy and mild cerebellar vermis hypoplasia.

**Case 121.83. Mutation c.811T>C (p.Trp271Arg) in *TBR1*.** The patient is currently 10 years old. She had severe developmental delay. She started to walk independently at the age of 7 years. She can eat with a spoon. She is nonverbal. She does not understand simple commands. She has poor eye contact, repetitive behavior and poor socialization and a diagnosis of autism. She has never had any developmental regression. She had a febrile status epilepticus at the age of 4 years and an unprovoked seizure at the age of 8 years. She was not treated with anticonvulsants and has not had any seizure recurrence yet. At 6.5 years, her weight was 17.7 kg (10^th^ percentile), height 107 cm (<3^rd^ percentile) and head circumference 51.6 cm (50^th^ percentile). She is not dysmorphic, has poor eye contact and her neurological examination is normal. Chromosomal microarray, karyotyping, methylation study of the genomic region associated with Angelman syndrome, mutation analysis of *MECP2* and *FOXG1*, Fragile X testing, and metabolic studies (plasma lactate and ammonia measurements, plasma amino acids and urine organic acids chromatographies, isoelectric focusing of serum transferring, plasma acylcarnitine profile) were normal. Brain MRI performed at the age of 1 year was normal aside from increased extra-axial spaces.

**Case 1464.524. Mutation c.511C>T (p.Gln171*) in *HNRNPU.*** This 4-year-old boy was born following an uneventful pregnancy and delivery. He has severe global developmental delay. He started to sit at the age of 22 months, crawl at 24 months. At the age of 4, he could stand with support and take a few steps with support. He did not have a pincer grasp but could reach for objects and bring them to his mouth. He does not understand simple commands. He does not have any words. He had some autistic features with poor eye contact and repetitive stereotypes movements. He had febrile seizures at he age of 12 months, then unprovoked generalized tonic-clonic seizures at the age of 18 months following which he was treated with clobazam and remains well-controlled, with one seizure per year on average. EEG was normal. On his most recent exam at the age of 4 years, his weight was 17.7kg (25-50^th^ percentile), height 96 cm (25th percentile) and head circumference 48.6 cm (25-50^th^ percentile). His eye contact was poorly sustained. He had a large forehead, apparent telecanthus and bilateral unilateral palmar creases. His tone was decreased centrally and in his limbs. Chromosomal microarray, Fragile X testing, and metabolic studies (plasma lactate, ammonia, and very long chain fatty acids measurements, plasma amino acids and urine organic acids chromatographies, isoelectric focusing of serum transferrin, urine purines/pyrimidines measurements) were normal. Brain MRI at the age of 18 months was normal.

**Case 762.297. Mutation c.263_266delAGAG (p.Glu88Glyfs*103) in *WAC.*** This 30-year-old female patient had an unremarkable perinatal history. She was hospitalized at the age of 10 months for investigation of hypotonia and developmental delay. She has moderate intellectual disability. She walked at the age of 24 months. She is able to read. She is shy but no behavioural difficulties. She has never had any seizures. On examination at the age of 23 years, her height was 157 cm (10-25^th^ percentile), her weight was 74.3 kg (75-90^th^ percentile) and head circumference was 53 cm (10-25^th^ percentile). She has minor dysmorphic features with prominent brow ridge and deep-set eyes. Her 5^th^ metacarpals were short. The remainder of her general and neurologic examinations was unremarkable. Chromosomal microarray, Fragile X, and metabolic studies (plasma amino acids and urine organic acids chromatographies) were normal. EEG at the age of 15 years revealed slow background but no epileptic abnormalities. Brain CT scan was normal.

**Case 341.162. Mutation c.14864G>A (p.Gly4955Glu) in *RYR2*.** This patient is a 9-year-old boy. He was the product of an uncomplicated pregnancy and delivery. He was globally developmentally delayed. He started to walk at the age of 3 years and said his first words around the age of 3 years. At the age of 7.5 years, he had 80 words and could make 2 to 3 word sentences. He had good social interactions. He had attention deficit and hyperactivity, but treatment with Ritalin did not result in any improvement. At the age of 22 months, he had 3 generalized tonic-clonic seizures for which he was hospitalized. Investigations at that time revealed an ectopic atrial tachycardia. He was started on clobazam and propranolol, which was then switched to metopralol. At the age of 4, he was again hospitalized for tachycardia and a secondary dilated cardiomyopathy. He was treated with amiodarone, digoxin, flecainide, nadalol and finally required the insertion of a cardiac pacemaker. He only had one seizure recurrence at the age of 9. He was operated for a unilateral cryptorchidism. On exam at the age of 7,5 years, his weight was 18 kg (<3^rd^ percentile), height 113.5 cm (<3^rd^ percentile), head circumference 49 cm [<3^rd^ percentile). He is not dysmorphic and his neurologic examination is unremarkable. Chromosomal microarray, Fragile X testing, methylation studies of the genomic region associated with the Angelman syndrome and metabolic studies (plasma lactate and ammonia measurements, plasma amino acids and urine organic acids chromatographies, urine purines/pyrimidines measurements, and isoelectric focusing of serum transferrin) were normal. Brain MRI and MR spectroscopy at the age of 2 years were normal.

**Case 1871.656. Mutation c.838C>T (p.Arg280Cys) in *MYH10.*** This is a two and a half year old girl with severe developmental delay, intractable epilepsy and microcephaly. She presented with infantile spasms and continues to have intractable epilepsy despite trial of multiple anticonvulsants that have included vigabatrin, ACTH, topiramate, leviteracetam, clobazam and lamictal. She is currently on clobazam, vigabatrin and lamictal and has daily seizures. Initially, she had classic flexor spasms, but now has more subtle seizure consisting of eye blinking. Her EEG showed a hypsarrythmic pattern initially, and now shows slow 1.5-2Hz bursts of focal and generalized spike and wave discharges with abnormal background. She is severely developmentally delayed. At the age of 2.5 years, she is unable to support her head, does not roll. She is able to smile. She has no words and does not understand. She is fed by gastrostomy. She has failure to thrive. She is cortically blind. On examination at the age of 2 years, she has mild dysmorphic features with long curly eyelashes, an upturned nose, and a pointed chin. Her head circumference is 41.5 cm (<2^nd^ percentile) and her weight is 8.5 cm (<2^nd^ percentile). She does not visually track nor does she blink to threat. She has some mild facial diplegia and important central and peripheral hypotonia. Chromosomal microarray, *CDLK5* sequencing, *MECP2* sequencing, *FOXG1* sequencing and deletion/duplication analysis, and metabolic studies (plasma lactate, ammonia and homocysteine measurements, plasma amino acids and urine organic acids chromatographies, urine creatine/guanidinoacetate and purines/pyrimidines measurements, plasma acylcarnitine profile, CSF neurotransmitters and pyridoxal-5-phosphate measurements) were normal. Brain MRI at the age of 5 months was normal. Repeat MRI brain at the age of 14 months revealed evidence of brain atrophy and increased T2 and FLAIR signal in the bilateral thalami, globi pallidi as well at the medial longitudinal fasciculus.

**Case 702.278. Mutation c.595G>A (p.Gly199Ser) in *EIF2C1.*** This female patient was born to an uncomplicated pregnancy and delivery. She had global developmental delay. She walked at the age of 24 months and said her first words at the age of 3 years. At the age of 12 years, she could speak in full sentences in both French and Arabic. She cannot read and can do only simple additions. Neuropsychology testing revealed a moderate intellectual disability. The patient presented with a status epilepticus with partial onset at the age of 8 years. She was treated with clobazam and remains well-controlled and seizure free. Her latest EGG at the age of 14 years revealed the presence of multifocal as well as generalized epileptic activity. The EEG also showed disturbance of the background rhythm. On exam at the age of 12.5 years, the patient had a height of 149.5 cm (25^th^ percentile), weight of 54.3kg (75-90^th^ percentile) and head circumference of 53.4 cm (50^th^ percentile). She was not dysmorphic. Her general and neurologic examinations were unremarkable. Chromosomal microarray, karyotyping, Fragile X testing and metabolic studies (plasma lactate and ammonia measurements, plasma amino acids and urine organic acids chromatographies, isoelectric focusing of serum transferrin) were normal. Brain MRI performed at the age of 10 years revealed increased T2 and FLAIR signal in the frontal periventricular regions.

**Case 1312.477. Mutation c.1111G>A (p.Gly371Arg) in *COL4A3BP.*** This patient is a 17-year-old male. Perinatal history was unremarkable. He had severe global developmental delay and intellectual disability. He walked at the age of 5 years. At the age of 17, he could say a few words and understand simple commands. He was starting to use utensils for feeding. He could not get dressed by himself. He had febrile seizures starting at the age of 3 years, then unprovoked focal motor seizures beginning at the age of 5 years, which were treated with clobazam. He had been off anticonvulsants and seizure-free for several years. He has episodes of agitation, auto-mutilation alternating with periods of apathy, which are treated with risperidone, paroxetine and valproate. He has a left renal agenesis and mild right vesiculouretral reflux. On exam at the age of 17, his weight was 53.7 kg (10^th^ percentile), height 163.7 cm (3-10^th^ percentile) and head circumference 57.3 cm (98^th^ percentile). He had poor eye contact. He had large distal phalanges but not dysmorphic. His examination was otherwise unremarkable. Chromosomal microarray revealed a 0.069 Mb duplication on chromosome 15q26.3 inherited from his phenotypically normal mother, thus was concluded to be of no clinical significance. *STXBP1* mutation analysis and metabolic studies (plasma lactate and ammonia measurements, plasma amino acids chromatography) were normal. Brain CT showed mild ventriculomegaly and mild periventricular leukomalacia.

**Case 115.81. Mutation c.699_701del (pTyr233*) in *SET*.** The patient is currently 12 years old. Pregnancy was unremarkable except for the detection of left renal agenesis on fetal ultrasound. Birth weight was 3.4 kg, and head circumference 32 cm (3^rd^ percentile). The patient had global developmental delay. He walked at the age of 27 months. He said his first words around the age of 4 years. At the age of 9 years, he could say 20 words, understand simple commands and ride a bicycle. He did not have any behavioral issues or any autistic features. He has moderate intellectual disability and is treated with Straterra for attention deficit without hyperactivity. On examination at the age of 9 years, his height was 121.3 cm (<3^nd^ percentile), weight 21.6 kg (<3^nd^ percentile) and head circumference 48 cm (< 3^rd^ percentile). Neurological examination is otherwise normal. Chromosomal microarray, karyotyping, Fragile X testing and metabolic studies (plasma lactate and ammonia measurements, plasma amino acids and urine organic acids, isoelectric focusing of serum transferrin) were normal. Brain MRI was normal.

**Case 670.267. Mutation c.1347_1348insA (p.Tyr450Ilefs92*) in *EGR1*.** This 5-year-old female patient was the product of an uneventful pregnancy and term delivery. She had a global developmental delay. She started to walk at the age of 18 months, could make 2-3 word sentences at the age of 5.5 years. She also has a motor apraxia and is treated for attention deficit and hyperactivity. She has no autistic features. She has never had any seizures. Her vision and hearing are normal. There is no developmental regression. On her latest examination at the age of 5 years and 5 months, she had an acquired microcephaly with a head circumference of 46.5cm (<3^rd^ percentile). Her weight was 16.8 kg (10-25^th^ percentile) and height 105.5cm (25^th^ percentile). She has no dysmorphic features. Her general and neurologic examinations were unremarkable. Chromosomal microarray, karyotyping, Fragile X testing, and metabolic testing (plasma lactate, ammonia and homocysteine measurements, plasma amino acids and urine organic acids chromatographies, urine purines/pyrimidines measurements, isoelectric focusing of serum transferrin) were normal. Brain MRI and MR spectroscopy performed at the age of 15 months was normal.

**Case 1439.518. Mutation c.909dupA (p.Tyr304Ilefs*19) in *PPP1CB.*** This 16-year-old female was born at term following an uneventful pregnancy. Delivery was by Cesearian section due to breech presentation. The patient was noted to be developmentally delayed at the age of 6 months. She walked at the age of 6 years. At the age of 13 years, she was nonverbal and could not communicate with a pictogram. She did not understand. She could feed herself with her fingers but not utensils. She did not have a social smile. There was no regression. She has never had a seizure. She has significant anxiety for which she takes buspirone. On exam at the age of 13, her weight was 26.4 kg (<3^rd^ percentile), height 134 cm (<3^rd^ percentile), and head circumference 52 cm (25^th^ percentile). She had a large mouth and malar hypoplasia. She had no eye contact and had repetitive stereotyped hand movements. Her tone was decreased in the limbs with brisk reflexes. Chromosomal microarray, methylation studies of the genomic region associated with Angelman syndrome and *MECP2* mutation analysis were normal. CT head revealed mild increased CSF spaces.

**Case 1841.646. Mutation c.413G>C (p.Arg138Pro) in *PPP2R2B.*** The patient is a 7-year-old boy. Prenatal and perinatal histories were unremarkable. He had a normal motor development but delayed fine motor and language. He said his first words at 2 years, and started to put 2 words together at 3.5 years. He has intractable epilepsy, with onset at the age of 3.5 years. He first presented with focal tonic seizures, then developed myoclonic, astatic and absence seizures. He has a diagnosis of Doose syndrome (myoclonic astatic epilepsy). The seizures are intractable to medication, which have included various combinations of clobazam, valproic acid, leviteracetam, ethosuximide and lamotrigine. He has a cluster of seizures every 2 to 3 months. He was hospitalized several times for status epilepticus. Psychology assessment at 4.5 years revealed moderate ID and a diagnosis of PDD-NOS. He has aggressive behaviour. On last examination at the age of 7 years, he was normocephalic (50-97th percentile). He had poor eye contact, but his general and neurologic examinations were otherwise unremarkable. Chromosomal microarray, SCN1A mutation analysis, and metabolic studies (plasma amino acids and urine organic acids chromatographies, urine creatine/guanidinoacetate and purines/pyrimidines measurements, isoelectric focusing of serum transferrin, plasma acylcarnitine profile, urine creatine/guanidinoacetate and purines/pyrimidines measurements, CSF glucose, neurotransmitters, and aminoacids measurements) were normal. Brain MRI and MR spectroscopy were normal.

**Case 580.240. Mutation c.286_287insC (p.Asn96Thrfs*35) in *CHMP2A.*** This 4-year-old boy was the product of an uncomplicated pregnancy and delivery. He was brought to medical attention at the age of 6 months for developmental delay. At the current age of 4 years, he is unable to sit or crawl. He can roll. He has no purposeful use of his hands. He tracks visually. His vision and hearing are normal. He smiles and understands some simple commands. He is nonverbal. He feeds by G-tube as he chokes on his secretions and has had multiple aspiration pneumonias. He developed partial complex seizures and generalized tonic-clonic seizures at the age of 4 years. He takes leviteracetam and has occasional breakthrough seizures. On examination at 4 years, his weight is 13.8 kg (10^th^ percentile), height 104.9 cm (50-70^th^ percentile) and head circumference 50 cm (25-50^th^ percentile). He is not dysmorphic. His tone is fluctuant, with decreased tone at rest. Chromosomal microarray, karyotyping, Fragile X testing, and metabolic studies (plasma lactate and ammonia measurements, plasma amino acids and urine organic acids chromatographies, urine purines/pyrimidines measurements, isoelectric focusing of serum transferrin) were normal. Brain MRI and MR spectroscopy were normal.

**Case 985.382. Mutation c.577_579delTCC (p.Ser193del) in *VPS4A*.** This 5-year-old boy was born to an uneventful pregnancy and delivery. His head circumference was 34 cm at birth. He has a severe global developmetal delay and acquired microcephaly. At the age of 5 years, he was unable to sit unassisted, and still had suboptimal head control. He could reach for objects. He could do a few signs. He was nonverbal. He was dependent in all activities of daily living. He did not have autistic features. He did not have any seizures. He has optic nerve hypoplasia noted on ophthalmologic exam. On exam, his head circumference was 40 cm (<3^rd^ percentile), height 91 c, (<3^rd^ percentile) and weight 11kg (<3^rd^ percentile). He is not dysmorphic. Scrotal hypoplasia is noted. He has significant central hypotonia and some spasticity in the four limbs. Reflexes were brisk. Chromosomal microarray, karyotyping and metabolic studies (plasma lactate, ammonia and very long chain fatty acids measurements, plasma amino acids and urine organic acids chromatographies, urine creatine/guanidinoacetate and purines/pyrimidines measurements, isoelectric focusing of serum transferrin) were normal. Brain MRI performed at the age of 4 months revealed evidence of increased CSF spaces, thinning of the corpus callosum and decreased white matter suggestive of cerebral atrophy.
